# Antisense Oligonucleotides Used to Target the *DUX4* mRNA as Therapeutic Approaches in FaciosScapuloHumeral Muscular Dystrophy (FSHD)

**DOI:** 10.3390/genes8030093

**Published:** 2017-03-03

**Authors:** Eugénie Ansseau, Céline Vanderplanck, Armelle Wauters, Scott Q. Harper, Frédérique Coppée, Alexandra Belayew

**Affiliations:** 1 Laboratory of Molecular Biology, Research Institute for Health Sciences and Technology, University of Mons, Avenue du Champ de Mars 6, 7000-Mons, Belgium; eugenie.ansseau@umons.ac.be (E.A.); Celine.Vanderplanck@sgs.com (C.V.); armelle.wauters@umons.ac.be (A.W.); frederique.coppee@umons.ac.be (F.C.); 2 Department of Pediatrics, The Ohio State University College of Medicine, Columbus, OH 43205, USA; scott.harper@nationwidechildrens.org; 3 Center for Gene Therapy, The Research Institute at Nationwide Children’s Hospital, Columbus, OH 43205, USA

**Keywords:** double homeobox, splicing interference, polyadenylation, primary myoblasts, myopathy

## Abstract

FacioScapuloHumeral muscular Dystrophy (FSHD) is one of the most prevalent hereditary myopathies and is generally characterized by progressive muscle atrophy affecting the face, scapular fixators; upper arms and distal lower legs. The FSHD locus maps to a macrosatellite D4Z4 repeat array on chromosome 4q35. Each D4Z4 unit contains a *DUX4* gene; the most distal of which is flanked by a polyadenylation site on FSHD-permissive alleles, which allows for production of stable *DUX4* mRNAs. In addition, an open chromatin structure is required for *DUX4* gene transcription. FSHD thus results from a gain of function of the toxic DUX4 protein that normally is only expressed in germ line and stem cells. Therapeutic strategies are emerging that aim to decrease DUX4 expression or toxicity in FSHD muscle cells. We review here the heterogeneity of *DUX4* mRNAs observed in muscle and stem cells; and the use of antisense oligonucleotides (AOs) targeting the *DUX4* mRNA to interfere either with transcript cleavage/polyadenylation or intron splicing. We show in primary cultures that DUX4-targeted AOs suppress the atrophic FSHD myotube phenotype; but do not improve the disorganized FSHD myotube phenotype which could be caused by DUX4c over-expression. Thus; DUX4c might constitute another therapeutic target in FSHD.

## 1. Introduction

### 1.1. Clinical Features of FSHD

FacioScapuloHumeral muscular Dystrophy (FSHD1, OMIM #158900) is an autosomal dominant hereditary myopathy with a strong epigenetic component that affects 1–9/100,000 [1], although a recent re-evaluation of FSHD frequency in The Netherlands suggested up to 12/100,000 births [2]. FSHD is among the most common diseases of skeletal muscles and involves muscle atrophy, inflammation and oxidative stress [3,4].

Muscle symptoms may appear during childhood but most patients manifest the disease in their second or third decade. Often, an asymmetric progressive muscle weakness begins with facial muscles causing eyelid drooping (ptosis), and an inability to whistle or smile [5]. These symptoms are missing in some patients. FSHD may also commonly affect the shoulder girdle leading to the inability to raise arms above the shoulder line [6]. Biceps, triceps, or deltoid muscles are unequally affected. Patients may also lose strength in abdominal muscles, which results in a protuberant abdomen and lordosis. Weakness in the lower legs commonly leads to foot drop, while involvement of other leg muscles may require wheelchair assistance (20% of patients).

One of the clinical hallmarks of FSHD is its heterogeneity ranging from severely affected wheelchair bound children to asymptomatic carriers in late adulthood. Disease progression (inflammation, atrophy and fat infiltration) in individual muscles can now be followed by magnetic resonance imaging (MRI) demonstrating the heterogeneity among patients [7,8,9].

Less commonly, FSHD patients may also show non-skeletal muscle phenotypes, such as high frequency hearing loss and retinal vasculopathy [10]. Although there is no major association with cardiomyopathy, about 10% of patients show an increased risk of arrhythmia [11,12].

### 1.2. The DUX4 Gene and Protein

The 4q35 genetic locus associated with FSHD has an unusual structure in that it comprises a polymorphic D4Z4 macrosatellite repeat array considered as “junk DNA” for a long time. The D4Z4 unit is 3.3-kb in length and belongs to a large family of repeat elements dispersed throughout the human genome such as on the short arms of the acrocentric chromosomes, and on chromosome 10q26 [13,14,15]. In 1994, an open reading frame (ORF) of a putative gene was mapped in the 4q35 D4Z4 unit. Its 3.3-kb sequence contained a predicted double homeobox, but neither a promoter nor a transcript had been found [16]. In a search for target genes of the Helicase-Like Transcription Factor (HLTF) we serendipitously identified a promoter inserted in the 5’ part of this ORF that reduced its size while maintaining the DoUble homeoboX (DUX) reading frame [17,18]. Because of their very high GC content several sequencing errors had occurred in the first publications on the characterization of these 3.3-kb elements [16,17,18]. Thus, based on these data, the D4Z4-resident ORF was thought to be a pseudogene and initially was not vigorously pursued as a potential FSHD gene. A 424-residue DUX4 protein of 52-kDa apparent molecular weight (MW) was later confirmed as the form expressed from the ORF; endogenous DUX4 in FSHD muscle cells appears slightly larger [19,20,21,22] most probably because of post-translational modifications.

The *DUX4* gene is a retrogene normally expressed in germ line and early embryonic cells, but suppressed by repeat-induced silencing in adult tissues [23]. However, it is activated in FSHD and expresses a double homeodomain transcription factor that binds to *cis*-elements containing two TAAT motifs typical of homeodomain binding sites [19,20,24]. Since some of its identified target genes encode transcription factors, DUX4 pathological activation leads to a large deregulation cascade and the main features of the disease. Forced DUX4 expression in muscle cells is toxic, leads to oxidative stress and interferes with myogenesis [20,21,22,25,26,27,28]. DUX4 expression results in the induction of several germline genes in FSHD muscles and in transfected cells [20,29]. DUX4 also transactivates retrotransposons and endogenous retrovirus elements, some of which constitute novel promoters for genes expressing proteins, long non-coding RNAs, or antisense transcripts. Many of these novel transcripts are expressed in FSHD muscle cells but not in control cells [30]. DUX4 is now recognized as the major cause of the pathology since activation of its target genes is the main molecular signature in FSHD muscle [29,31].

Besides its transcriptional activity, DUX4 disrupts RNA metabolism including RNA splicing, nonsense mediated RNA decay and transport, microRNA processing, but also nuclear bodies, cell signaling, polarity and migration pathways [32,33,34,35]. DUX4 interferes with quality control not only of RNAs but also of proteins; for example, it inhibits protein turnover and induces nuclear aggregation of TAR DNA-binding protein 43 (TDP-43), a transcriptional repressor. Similar pathological changes are found in diseases such as amyotrophic lateral sclerosis (ALS) and inclusion body myopathy [36].

DUX4c is a 374-residue homologous protein encoded by a truncated inverted D4Z4 element mapped 42 kb centromeric of the D4Z4 repeat array. DUX4 and DUX4c share a high sequence similarity with identical double homeodomain but diverge at the carboxyl-terminal domain. DUX4c is expressed at low level in healthy muscle cells and is up-regulated in FSHD; it favors myoblast proliferation and inhibits their differentiation [26,37]. Our group recently showed that DUX4 and DUX4c translocate from the nucleus to the cytoplasm at the time of myoblast fusion [38]. A search for DUX4/4c partners identified desmin and other cytoskeleton-associated proteins as well as RNA binding proteins involved in splicing and translation. Fused in Sarcoma (FUS) is a partner that shares normal and pathological functions with TDP-43 such as the formation of pathological aggregates in ALS degenerating motor neurons [36]. FUS and other partners associate with IGF2 mRNA binding proteins and belong to ribonucleoparticle (mRNP) granules that carry translationally repressed mRNAs (such as beta actin mRNA) along microtubules for translation at specific sites and times during cell differentiation [38].

### 1.3. The Genetic and Epigenetic Conditions Required to Develop FSHD

FSHD is an unusual pathology in that its development requires both genetic and epigenetic conditions. The genetic condition is the presence of a complete *DUX4* gene. Every D4Z4 element contains a promoter and the DUX4 ORF, but lacks a polyadenylation signal (PAS) resulting in a rapid *DUX4* mRNA degradation. In contrast transcripts initiated in the distal D4Z4 unit on a 4qA permissive allele extend outside of the repeat array and reach a PAS in the flanking pLAM sequence [19,39]. The resulting poly-A tail stabilizes the *DUX4* mRNAs and allows for their translation into a toxic protein. Other mechanisms might provide a PAS to the *DUX4* gene. In a patient with FSHD, a 4/10 chromosome rearrangement brought the pLAM PAS at the end of the homologous repeat array on chromosome 10q26; this repeat array is normally non-pathologic but the PAS translocation caused 10q-associated *DUX4* mRNA stabilization [39].

The epigenetic condition is an open chromatin structure allowing the transcriptional apparatus accessibility to the *DUX4* gene. The 4q35 D4Z4 repeat array is normally associated with heterochromatin, resulting in very low or absent *DUX4* transcription. In FSHD, this region undergoes epigenetic alterations (DNA hypomethylation) following either contraction of the D4Z4 repeat array (FSHD1: OMIM #158900; 95% of cases) or by mutation in a chromatin organizer such as the *SMCHD1* [40] or *DNMT3B* [41] genes (FSHD2: OMIM #158901; digenic inheritance pattern). Both a full *DUX4* gene and its presence in an open chromatin structure are required for FSHD development (reviewed by [42,43,44]).

The large clinical heterogeneity in age of onset and disease progression can now largely be accounted for by epigenetic instability. Patients with FSHD1 and D4Z4 repeat arrays of 1–6 units usually present a clinical severity related to the array shortening that is itself correlated with DNA hypomethylation. Individuals with 7–10 repeat units appear more susceptible to epigenetic variations resulting in a severity range from non-penetrance when D4Z4 DNA is highly methylated, to serious presentation when D4Z4 DNA has a very low methylation [45,46,47]. The role of DNA hypomethylation is exemplified by the high clinical severity observed in patients who inherited combined FSHD1 and 2 defects [48,49]. Besides SMCHD1 and DNMT3B role in DNA methylation, telomere shortening with ageing [50], proteins of the Polycomb (inhibitors) or Trithorax (activators) family, a long non-coding RNA [51], and several antisense transcripts [22] can also affect D4Z4 epigenetics in FSHD cells and additional modifying factors are expected to be identified [43].

### 1.4. Therapeutic Approaches

As no curative treatment for FSHD is available, clinical management involves physical therapy, aerobic exercise, respiratory function therapy, and orthopedic interventions [52,53,54]. Several studies indicated that a specific oxidative stress was part of the FSHD pathology, and could result from DUX4 expression [4,25,55]. A randomized, double-blind, placebo-controlled pilot clinical trial was set up involving oral administration of vitamins C and E, zinc gluconate, and seleno-methionine. Following 17-week supplementation, most patients presented a higher strength and endurance of the quadriceps and a decrease in oxidative stress blood markers [56]. This approach is expected to stabilize or slow down disease progression, but several therapeutic strategies aiming for the molecular causes of FSHD (chromatin opening, *DUX4* gene expression) are in development [57,58,59,60]. Interestingly, 60% of the DUX4 toxicity inhibitors identified in a high throughput screen protected myoblasts from oxidative stress inducers [61].

In contrast to Duchenne Muscular Dystrophy (DMD), which results from loss of function mutations in the dystrophin gene, FSHD is linked to a gain of function of the DUX4 protein in skeletal muscles. Thus, if antisense strategies are designed to restore the dystrophin reading frame in DMD (reviewed by [62,63,64]) they aim to decrease or suppress DUX4 in FSHD. In the present article we review the use of antisense oligonucleotides (AOs) targeting the *DUX4* mRNA as therapeutic agents in FSHD.

## 2. Material and Methods

### 2.1. Ethics Statement

Primary human myoblasts were derived from muscle biopsies performed according to the current ethical and legislative rules of France, and written informed consent was obtained from all subjects, as directed by the ethical committee of the Centre Hospitalier Universitaire (CHU) Arnaud de Villeneuve (Montpellier, France) [65,66]. In addition, the use of this material was approved by the ethics committee of the University of Mons (ref # A901) and the ethics committee of ULB-Erasme (Brussels ref #B2011/003 and #P2015/516).

The clones of immortalized myoblasts were derived from a mosaic individual [67], and kindly provided by Profs. S. Van der Maarel and G. Butler-Browne.

### 2.2. Myogenic Cell Culture

Primary human myoblasts from an unaffected control and a patient with FSHD were isolated from muscle biopsies, purified and established as described previously [65,66]. The myoblasts were grown in 35 mm collagen-coated dishes (Ywaki, Tokyo, Japan) in DMEM with 4.5 g/L glucose and l-glutamine (Lonza, Verviers, Belgium) as well as gentamycin (50 µg/mL, Sigma-Aldrich, Gillingham, UK), 10% fetal bovine serum (Invitrogen/Thermo-Fisher Scientific, Waltham, MA, USA ), and 1% Ultroser G (Pall BioSepra, Cergy-St-Christophe, France) at 37 °C under 5% CO_2_. Confluent myoblast cultures were differentiated by switching the medium to DMEM/gentamicin (50 µg/mL) with 2% FBS.

The immortalized myoblasts were grown and differentiated as described [67].

### 2.3. siRNA and AOs Transfection

The custom siRNA targeting DUX4 obtained from Ambion/Thermo-Fisher Scientific, and the cell transfection conditions were previously described [27]. We used the “Silencer siRNA Starter Kit” (Ambion/Thermo-Fisher Scientific) with the “SiPORT NeoFX” transfection agent and used 4 µL of siPORTNeoFX and 10 nM siRNA for primary human myoblasts.

The custom AOs and the cell transfection conditions were previously described [27]. We used Fugene HD with either the negative control AO mGMCSF3A (−5 + 20) (nc-AO, 600 nM) or the indicated DUX4-AOs at an optimized concentration. All transfections were performed in duplicate wells and were repeated 3 times to ensure consistency.

### 2.4. Immunofluorescence

Human primary myoblasts were fixed in PBS containing 4% paraformaldehyde (Sigma-Aldrich) and treated with PBS/0.5% Triton X-100. After blocking in PBS/20% FBS, the cells were incubated with primary antibodies for 2 h at room temperature. The following antibodies and dilutions were used: mouse monoclonal (mAb) anti-troponin T 1/100 (clone JLT-12, Sigma-Aldrich), and mAb 9A12 (which we developed against DUX4) 1/50 [19]; clone 9A12, Merck Millipore, Darmstadt, Germany). After washing and blocking, cells were incubated for 1 h at room temperature with Alexa Fluor secondary antibodies at 1/100 (goat anti-mouse 488 and anti-rabbit 555, Invitrogen), then washed again and mounted with Vectashield mounting medium containing DAPI (Vector Laboratories, Burlingame, CA, USA). Images were acquired with a fluorescence microscope (Olympus, Tokyo, Japan).

### 2.5. RNA Analysis

Total RNA was extracted from different myoblast cultures and 3’RACE performed as described [27]. The full DUX4 ORF was specifically amplified by RT-PCR as described [19].

For RT-qPCR, cDNA was synthesized with 1 µg of total RNA and the Maxima First Strand cDNA Synthesis Kit (Thermo Fisher Scientific,). All qPCRs were performed in triplicate. We used SYBR green master mix of GoTaq qPCR Systems (Promega, Madison, WI, USA ) and 0.3 µM of each primer except for DUX4_Rev at 0.9 µM (DUX4 primers (Forward: 5′ ACTGCCATTCTTTCCTGGGCAT 3′; Reverse: 5′ GGGAGACATTCAGCCAGAATTTC 3′); TRIM43 primers [67]; RPLPO primers (Forward: 5′ TCATCCAGCAGGTGTTCG 3′; Reverse: 5′ AGCAAGTGGGAAGGTGTAA 3′; [68]. For gene expression analysis, 3 µL of diluted cDNA was used per reaction on a StepOnePlus System (Applied Biosystems/ Thermo-Fisher Scientific). Cycling conditions were as follows: initial denaturation step at 95 °C for 3 min, followed by 40 cycles of 10 s at 95 °C and 60 s at 60 °C, for DUX4 amplification the annealing temperature is 62 °C. The specificity of all the reactions was monitored by a melting curve analysis. The data were analysed with the StepOnePlus software. The relative expression was calculated, using RPLPO mRNA as a reference for cDNA input because it was shown to be stable during muscle differentiation [68] and following M. Pfaffl’s guidelines [69].

### 2.6. Statistical Analyses

Statistical significance was evaluated using Student’s *t*-test. A *p* value < 0.05 * was considered significant.

## 3. Results and Discussion

We have first aligned the structures of the different *DUX4* mRNAs that have been characterized to-date. We shall then summarize the strategies established to interfere with either processing of the mRNA 3’ end or pre-mRNA splicing, and the regions that were targeted to develop specific AOs. Finally, we shall present data that indicate DUX4 is not the only target gene to be considered in treating FSHD.

### 3.1. The Heterogeneity of DUX4 Transcripts

**Figure 1:**
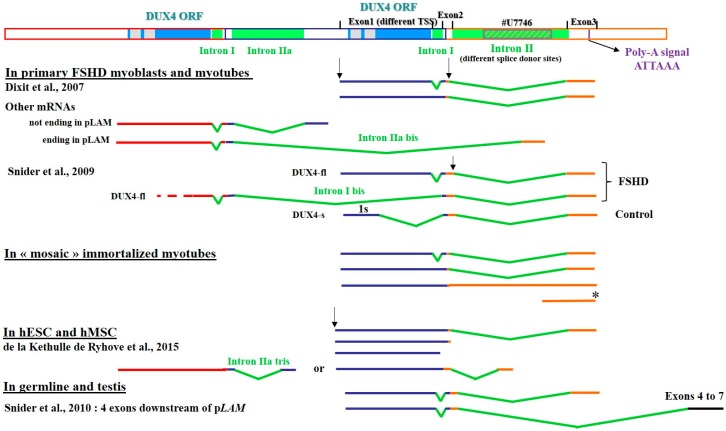
*DUX4* mRNA variants. Schematic representation of the D4Z4 repeat array (Genbank # AF117653), the last D4Z4 unit and the adjacent pLAM region (Genbank #U7746). The *DUX4* ORF is contained in the first exon. Two polyadenylation signals (PAS) were reported, one in exon 3 and one in exon 7 [19,23]. All the mRNAs reported to date or identified in our group and not published yet are represented as detected in primary FSHD myoblasts/tubes [19,22], in immortalized myotubes from clones of a mosaic individual, in human Mesenchymal Stromal cells [70] and in germline and testis [23]. We observed two different transcription start sites (TSS) and different splice donor sites for intron II. These mRNAs derived exclusively from chromosome 4. The short mRNA (*) found in FSHD immortalized myotubes is discussed in the text.

A major difficulty in the study of endogenous *DUX4* gene expression is the very low abundance, high GC content and rapid turnover of its mRNAs. Despite these challenges, investigators of different groups have characterized several transcription starts sites, alternative splicing and use of polyadenylation signals, as well as additional exons in the 3’ untranslated region (3’ UTR) in different cell types. Some of these variations result in the expression of DUX4 protein forms either shorter [22,23] or extended on the amino-terminus [70] as compared to the *bona fide* 424-residue protein described in muscle cells. In Figure 1 we have summarized the different characteristics of these *DUX4* mRNAs.

In FSHD primary muscle cells an alternative splicing of intron I was identified downstream of the stop codon, thus not affecting the protein sequence. In healthy control muscles *DUX4* mRNA levels are much lower and another splicing event was described that could lead to the expression of a shorter protein missing the carboxyl-terminal part outside of the homeodomains. This led to the distinction between full length (DUX4-fl, 424 residues) and short (DUX4-s, 161 residues) proteins [22,23]. All those mRNAs present a micro-heterogeneity in the 3’ end, with 3 cleavage sites located 16 to 22 nucleotides (nt) downstream from the PAS [71].

As transcription could initiate in each D4Z4 element [19] and because of the repetitive nature of the array, some *DUX4* mRNAs could extend across several D4Z4 units and end up at the PAS in the pLAM region. These RNAs could be submitted to complex splicing processes resulting in truncated DUX4 ORFs. Several examples of such RNA sequences are shown in Figure 1 (other mRNAs).

We did not find any PAS for those mRNAs. They ended in intron II at positions 10,450 or 10,551 (GenBank #AF117653) or at the homologous positions in the pLAM sequence (GenBank #U74497). In those mRNAs, intron I was spliced out as usual and the alternative intron IIa was comprised between positions 9045 to 10,085. Such mRNAs are rare but observed in several experiments and in different cell types. In addition to the primary FSHD myotubes we detected such splicing in control cells such as immortalized myoblasts (data not show in this paper) and embryonic stem cells (bottom of Figure 1). In ES cells, this mRNA ended at position 10,224, and an alternative intron II (named intron IIa tris) mapped between positions 9045 to 9840.

Myoblasts grown from a biopsy of a mosaic individual were immortalized by transduction with a recombinant virus expressing the hTERT telomerase gene. The resulting individual clones are genetically identical, except for the size of the D4Z4 repeat array, that is either large (control) or contracted as in FSHD1 [67].Total RNA was extracted from immortalized FSHD (54-12; 54-2) or control (54-6; 54-A1) myotubes at 5 days of differentiation. We performed 3’RACE as described [27] and analyzed the resulting products by agarose gel electrophoresis (Supplementary Materials Figure S1A). In the FSHD clones (54-12 and 54-2) we detected three bands at 400 bp, 550 bp and at 900 bp. Based on sequence determination, those bands respectively corresponded to *DUX4* mRNA with either intron I and II spliced out, intron II only spliced out or the unspliced form. Those three RNA types were also detected in control clones (54-6 and 54-A1) but at a very low level.

In addition, we performed an RT-PCR to specifically amplify the full DUX4 ORF as described [19] (Supplementary Materials Figure S1B). We detected a 1700-bp product in FSHD clones 54-12 and 54-2 but at much lower abundance in the latter as published [67]. This product was cloned and based on sequence determination, corresponded to *DUX4* mRNA with only intron II spliced out. It was not detected in the control clones. A 450-bp RT-PCR product was obtained with FSHD RNA that corresponded to a small ORF present in the pLAM region (see below, * on Figure 1). This ORF begins 188 nt 3’ from the end of intron II, contains only the beginning of the first DUX4 homeobox and ends up at a stop codon 28 nt further downstream in exon 3.

As shown by the agarose gel electrophoresis (Supplementary Materials Figure S1), the most abundant *DUX4* RNA form is the one where only intron II is spliced out. Very few RNAs present both intron I and II either spliced out or unspliced.

*DUX4* mRNAs are also detected in testis, in healthy human germline [23], and embryonic and mesenchymal stem cells (hESC and hMSC) [70]. Intriguingly upon osteoblastic differentiation MSC express the *DUX4* gene from alternative upstream promoters resulting in proteins extended on the amino-terminus, one of which corresponds to the initial ORF described by Hewitt et al. [16]. Such extended mRNAs were detected in hESCs and hMSCs [70]. Most of these mRNAs presented 3’ ends typical of FSHD myoblasts (primary or immortalized) but some ended just 5’ of intron II or within intron I. Those shorter forms still encompassed the *DUX4* ORF. Moreover, longer *DUX4* mRNAs were described in healthy testis and germline extending to four additional exons downstream of pLAM. In germline the mRNAs contained either exons 1, 2 and 3, or exons 1, 2, 6 and 7. In testis the mRNA encompassed exons 1, 2, 4, 5, 6 and 7. If the sequences of exons 4 and 5 corresponded to chromosome 4, intriguingly the sequences of exon 6 and 7 were from chromosome 10. Obviously, for these longer RNAs another PAS located in exon 7 is used [23].

### 3.2. Defining Targets for Antisense Oligonucleotides on DUX4 mRNAs

The *DUX4* coding sequence is entirely located in the gene first exon. Thus, a disruption of its reading frame by antisense-mediated exon skipping could not be considered. However, several researchers decided to target elements in the mRNA 3’ UTR to either disrupt the permissive polyadenylation or interfere with intron 1 or 2 splicing. Since *DUX4* mRNA and protein levels are low in FSHD muscle cells and difficult to detect, researchers often quantify the mRNAs of DUX4 target genes (named “footprint genes”) as biomarkers of its activity/presence [20].

#### 3.2.1. Interference with mRNA Cleavage and Polyadenylation

Because the only stable *DUX4* mRNAs are the ones extended with a poly-A tail typically transcribed from the 4qA permissive allele, several groups considered interfering with transcript termination and 3’ end processing to cause mRNA degradation and decrease DUX4 protein expression. The RNA polymerase II complex produces a primary transcript extending across the PAS and that is processed by the Cleavage and polyadenylation multiprotein complex composed of cleavage factors, cleavage stimulation factors, cleavage and polyadenylation specificity factors and nuclear poly-A polymerases [72]. The primary transcript is normally cleaved 10–30 nt downstream from the PAS which has the sequence AUUAAA derived from the 4qA permissive allele. It requires binding of the Cleavage stimulation Factor (CstF) onto a U/GU-rich sequence (Downstream Sequence Element, DSE) located 30–60 nt 3’ of the PAS (for review see [73]. The 3’ end processing occurs co-transcriptionally and favors mRNA nuclear export, stability, and translation and thus constitutes a target for suppression of gene expression.

Two research groups independently developed AOs to target *DUX4* pre-mRNA elements involved in its cleavage or polyadenylation in order to suppress the protein expression [71,74]. They selected the phosphorodiamidate morpholino (PMO) chemistry for its lack of toxicity in clinical trials for DMD. Among the different PMOs, the most efficient targeted the PAS on *DUX4* mRNA, and its 25-nt sequence turned out to be identical for both groups, and was also found in our study of a larger AO set (see below). DUX4 suppression was evaluated either by quantitation of several of its footprint gene mRNAs that constitute FSHD biomarkers [71] or by a differential transcriptome study (RNA-seq) of FSHD or healthy myoblasts treated or not with the PMO [74]. This RNA-seq study indicated that 96% of the genes upregulated in FSHD myotubes were at least partly reduced by the PAS-PMO, while 89% of the mRNAs that were significantly reduced had a least some upregulation in FSHD myotubes. The inhibition was dose-dependent with 50% reduction of footprint gene expression in the 1–3 µmol/L PMO concentration range [74]. The inhibition was also obtained in a model of FSHD muscle xenograft in an immunosuppressed mouse with electroporation-mediated PMO administration [74].

A difficulty in such an approach is the recently discovered heterogeneity of polyadenylation sites that suggests the *DUX4* mRNA could use alternative PAS if the major one on 4qA becomes unavailable [75,76]. Alternative polyadenylation generates 3′ UTRs of different lengths: these can recruit different factors that can impact on mRNA localization, mRNA and protein abundance, and protein intracellular location [77,78].

#### 3.2.2. Interference with mRNA Splicing

##### Muscle Cells

**Figure 2:**
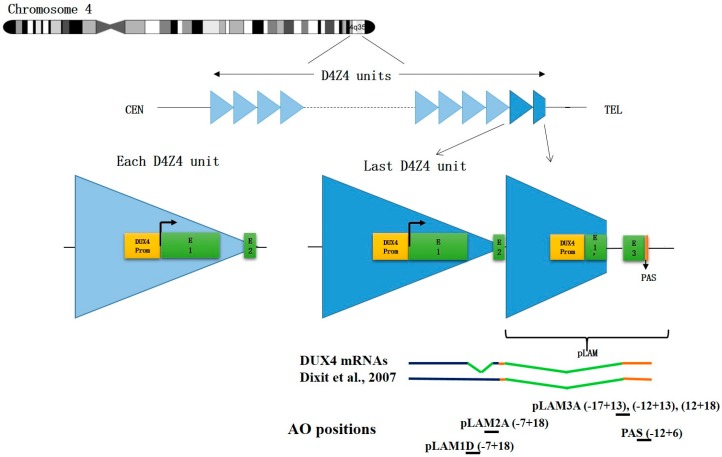
D4Z4 repeat array on chromosome 4 and antisense oligonucleotide positions. **Top:** Each D4Z4 repeated unit contains a promoter and an open reading frame (ORF) for DUX4 in the first exon (E1) and a short non-coding exon 2 (E2). On a 4qA permissive allele, the last D4Z4 unit is extended by a pLAM region providing intron I, an untranslated exon 3 (E3) and a polyadenylation signal (PAS) allowing for *DUX4* mRNA stabilization and subsequent translation in DUX4 protein. Healthy individuals present 11–100 D4Z4 units while patients with FSHD1 only have 1–10 units. **Bottom:** Positions of the antisense oligonucleotides (AOs) described in this review. They are all designed for splicing interference except for PAS that affects mRNA polyadenylation.

AO-mediated splicing interference is used to induce exon skipping on a pre-mRNA allowing for the expression of a missing protein as done for DMD and spinal muscular atrophy (SMA) therapy (see reviews by [62,79]). Alternatively AOs targeting splice sites can destabilize an mRNA and create a transient phenocopy of a loss of function mutation [62]. It is in that perspective that in collaboration with Prof. Steve Wilton (Centre for Comparative Genomics, Murdoch University, Australia, and Centre for Neuromuscular and Neurological Disorders , University of Western Australia) our group designed AOs complementary to donor and acceptor splice sites of exons 2 and 3 both located in the 3’UTR of the *DUX4* pre-mRNA [27] (Figure 2). This region is the most different from the homologous DUX4c sequence. The AOs were 25–30 nt oligomers synthesized as 2′-*O*-methyl modified riboses linked by phosphorothioates.

The splice-switching efficacy of these AOs on *DUX4* mRNA and their specificity towards homologous DUX4c mRNA were evaluated by co-transfection of C2C12 mouse myoblasts with expression vectors containing a *CMV* promoter linked to either *DUX4* exons 1, 2 and 3 (*pCIneo-DUX4)* or the *DUX4c* gene (*pCIneo-DUX4c).* The DUX4 and DUX4c proteins were immuno-detected (western blot) in extracts of cells harvested 24 h post transfection: an AO concentration range was evaluated for suppression of DUX4 but not DUX4c protein. AOs pLAM2A(−7 + 18) and pLAM3A(−12 + 13) presented the highest efficacy at concentrations of 50 and 10 nM respectively. The biological impact of these AOs was demonstrated by decreases in MURF and Atrogin-1 (linked to muscle atrophy) and TP53, all proteins induced by DUX4 expression and part of its deregulation cascade.

These AOs were further evaluated on endogenous DUX4 expression in primary FSHD myoblasts. The cells were switched to differentiation medium 4 h after transfection with the AOs to induce endogenous DUX4 and DUX4c expression, and the resulting myotubes were lysed three days later for protein and RNA extractions. DUX4 protein levels that are pretty low in primary myotubes (expression in 1/200 myonuclei) [80] could not be detected anymore with either AO. Based on RT-PCR the *DUX4* mRNAs were decreased about 50% with pLAM3A(−12 + 13) that interferes with intron II splicing but only 30% with pLAM2A(−7 + 18) which targets the alternatively spliced intron I. The reduction in TP53 amounts was stronger with intron II than intron I splicing interference suggesting residual DUX4 protein amounts undetected by our western blot analysis were still expressed from the alternatively spliced mRNA. In the study by Chen et al. [74] a splice switching AO was also tested but found to be less efficient at DUX4 suppression than the PAS-targeted AO. The targeted splice site was in intron I that is only alternatively spliced explaining that not all mRNA molecules were affected like we had observed [27].

**Figure 3:**
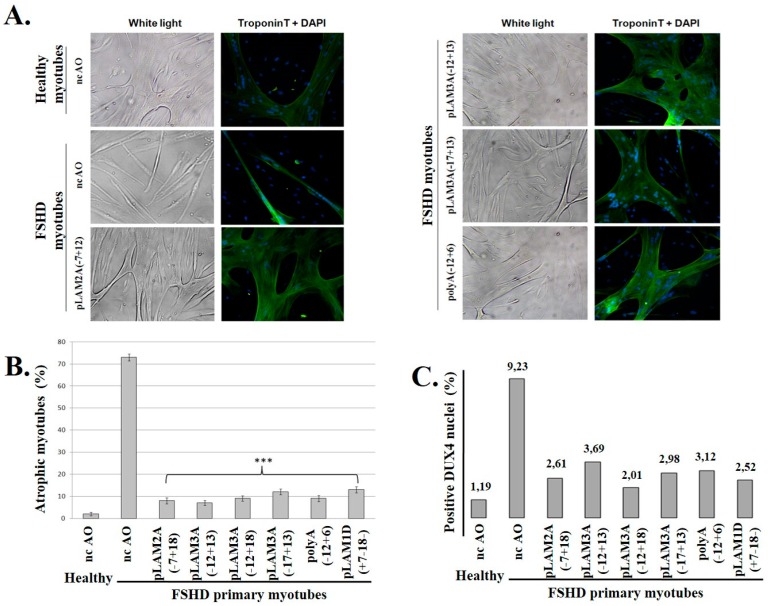
Treatment of FSHD myoblasts with antisense oligonucleotides against DUX4 prevents the formation of myotubes with the atrophic phenotype. (**A**) Primary healthy or FSHD myoblasts were transfected with either the negative control AO (nc-AO) or the indicated AOs targeting *DUX4* mRNAs (AO positions shown in Figure 2), and differentiation was induced 4 h later. After 8 days, cells were observed under white light before processing to detect troponin T through immunofluorescence (green). The nuclei were stained with DAPI; (**B**) The efficacy of the AOs used in (**A**) and two additional ones was evaluated based on the proportion of atrophic myotubes (caused by DUX4 expression, and observed in the ncAO-treated cells) in the culture. Myotubes were counted from at least 10 random fields: those with a width <20 µm were considered “atrophic”. The percentage of atrophic to total myotubes is expressed as the mean ± SD; (**C**) DUX4 was detected by immunofluorescence (MAb 9A12; not shown). The number of DUX4-positive nuclei was counted in 10 fields in healthy control or FSHD myoblast cultures following treatment with the indicated AOs. The percentage of DUX4-positive nuclei among total nuclei (DAPI staining) was calculated and is reported in the graph. The significance of the differences between experiments with each individual AO compared to the ncAO, was evaluated using Student’s *t*-test. *** *p*-value < 0.001.

We then compared the impact on FSHD myotubes of a set of AOs directed against different regions of the *DUX4* pre-mRNA. The transfection was done on FSHD myoblast cultures that were switched to differentiation medium 4 h later. The morphology of the resulting myotubes was analyzed 8 days later following immunofluorescence staining of Troponin T (Figure 3). Endogenous DUX4 expression induces an atrophic phenotype (myotube width < 20 µm [27] as observed here in culture dishes treated with the negative control nc-AO (Figure 3A left panel). The FSHD atrophic myotubes became larger when DUX4 was suppressed (Figure 3B).

The ability of each AO to prevent DUX4 expression was evaluated by counting the percentage of DUX4-positive nuclei following immunofluorescence staining with a specific monoclonal antibody (9A12) (Figure 3C and Supplementary Materials Figures S2). All the tested AOs could decrease the percentage of DUX4-positive nuclei 3- to 4.5-fold as compared to the nc-AO, and reduce 6- to 8-fold the number of atrophic myotubes in the culture.

##### Human Mesenchymal Stromal Cells and Embryonic Stem Cells

In a study on osteoblastic differentiation of human mesenchymal stromal cells (MSCs), isolated from bone marrow in collaboration with Dr. L. Lagneaux (Bordet Institute, ULB, Brussels, Belgium) our group identified several putative DUX4 protein forms (58- and 70-kDa besides *bona fide* 52-kDa DUX4) in western blots probed with the specific monoclonal antibody (9A12) we had developed [19]. A switch occurred between days 7–8 of differentiation from the expression of the 52- to the 58-kDa form that became very abundant while the 70-kDa form remained constant. We transfected MSCs with an AO interfering with splicing of intron I (pLAM1D) to evaluate whether *DUX4* mRNA destabilization affected the expression of those proteins, and could thus confirm the nature of 52- and 58-kDa DUX4. The characterization of *DUX4* mRNAs defined an upstream start site in human embryonic stem cells with the FSHD1 defect, extending the ORF with 60 codons, thus corresponding to the one initially defined by Hewitt et al. [16] and encoding 58-kDa DUX4. Gain and loss of function experiments demonstrated opposite roles for the DUX4 forms in osteoblastic differentiation of MSCs as evaluated from measures of alkaline phosphatase activity and calcium deposition: it was favored by 52-kDa DUX4 but delayed by 58-kDa DUX4 expression. Optimal differentiation required a precise balance between both DUX4 forms [70]. Since both mRNAs were destabilized by the AOs developed against FSHD, caution should be exerted in their use to avoid suppressing a normal DUX4 function in MSCs. A muscle-specific delivery should be considered.

### 3.3. DUX4 Inhibition Prevents the Formation of Atrophic, but Not Disorganized FSHD Myotubes

FSHD primary myotubes present either a narrow elongated shape with well aligned nuclei (atrophic phenotype) or giant disorganized structures with large clusters of nuclei (disorganized phenotype). Each culture presents a mix of either myotube phenotype in different proportions [65,66]. We selected two FSHD primary myoblast lines that were previously shown to mostly differentiate into myotubes with either the atrophic (aFSHD) or disorganized (dFSHD) phenotype.

**Figure 4:**
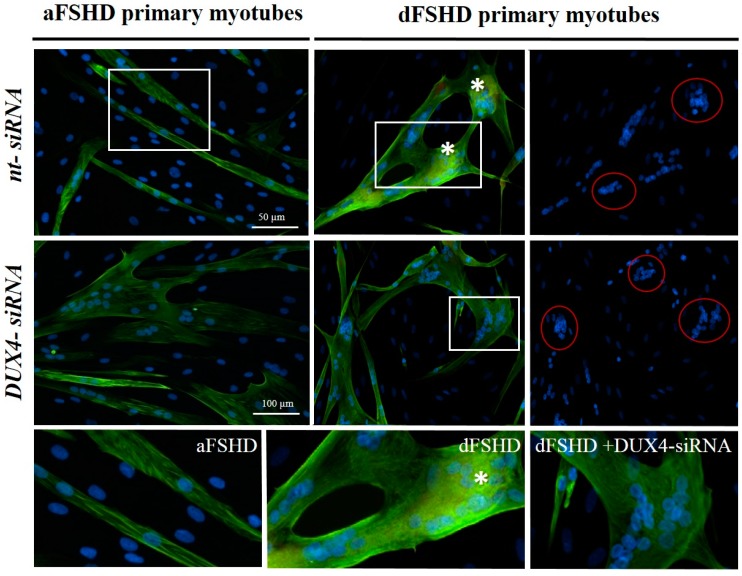
DUX4 inhibition prevents the formation of atrophic, but not disorganized FSHD myotubes. aFSHD and dFSHD primary myoblasts were transfected with either DUX4 siRNA or a non-targeted (nt) siRNA as indicated and switched to differentiation medium. Eight days later, the myotube morphology was highlighted through immunofluorescence staining of troponin T (green) and nuclei with DAPI (blue). Bottom panels: Magnified boxed regions. Stars indicate the accumulation of troponin T near clusters of nuclei. Scale bars: 50 µm.

We transfected these myoblasts with short inhibitory RNAs, either DUX4 siRNA or a negative control siRNA (nt) and switched the culture to differentiation medium. Eight days later, the myotube morphology was highlighted through immunofluorescence staining of troponin T, a cytoplasmic marker of muscle differentiation. As expected, aFSHD myoblasts differentiated into atrophic myotubes in the presence of negative control siRNA. In contrast, the myotubes that formed after treatment with DUX4 siRNA (Figure 4, left panels) were enlarged. Similar suppression of the atrophic myotube phenotype was previously observed using this siRNA in healthy myoblasts transfected with a DUX4 expression vector [27]. However, in the presence of either negative control or DUX4 siRNA, dFSHD myoblasts differentiated into disorganized myotubes with large clusters of myonuclei (Figure 4, right panels), suggesting that this abnormal phenotype did not result from DUX4 expression.

**Figure 5:**
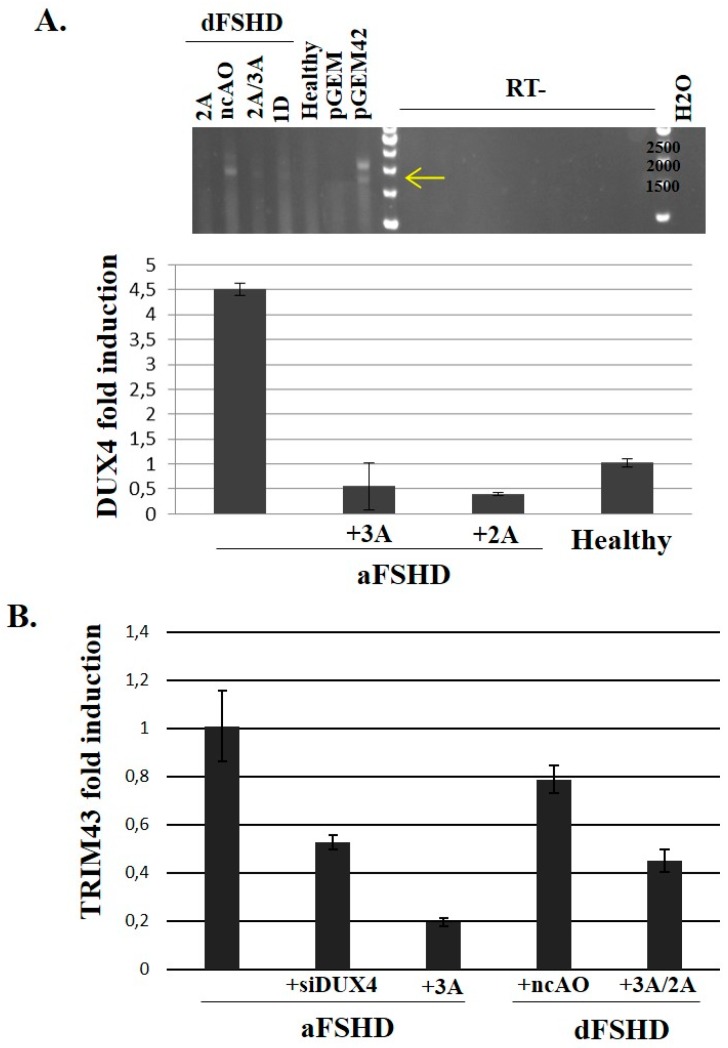
Evaluation of DUX4-AO efficiency on endogenous DUX4 and target mRNA expression in FSHD primary myotubes. 10^5^ primary myoblasts (control, aFSHD and dFSHD) were seeded in 35 mm culture dishes. The next day, cells were transfected with either the negative control AO (nc-AO, 600 nM) or AOs pLAM2A(−7 + 18) and pLAM3A(−12 + 13) either alone (lanes 2A or 3A) or in a cocktail (lane 2A/3A) and pLAM1D(+7 − 18) (lane 1D) at previously determined optimal concentrations. Differentiation was induced 4 h after transfection and the cells were harvested 72 h later. (**A**) **Top:** Total RNAs of dFSHD myotubes were extracted and submitted to RT-PCR with primers we had previously shown to be specific of the DUX4 full length ORF [19]. The RT-PCR products were separated by electrophoresis on an agarose gel and stained with ethidium bromide. The controls were total RNAs of C2C12 cells transfected with the pGEM plasmid either without insert (negative control) or with a genomic fragment containing 2 D4Z4 units [18] (*pGEM42*). The experiment was done in the presence (RT+) or absence (RT-) of retrotranscriptase to demonstrate the products did not result from amplification of contaminating genomic DNA. **Bottom:** Total RNAs of aFSHD myotubes were extracted and submitted to reverse transcription (RT) and amplification by qPCR with DUX4-specific primers. The relative abundance was calculated using Ribosomal Protein Lateral Stalk Subunit P0 (RPLPO) as a reference for cDNA input and following M. Pfaffl’s guidelines [68,69]. The data are presented as fold change in *DUX4* mRNA abundance with or without AO treatment; (**B**) Fold change in TRIM43 mRNA abundance after treatment with an AO or siRNA against *DUX4* mRNA. The RT-qPCR was performed as described [67].

To evaluate the impact of the AO treatments on *DUX4* mRNA levels in dFSHD cells, we extracted total RNAs from aFSHD and dFSHD primary myotubes and performed an RT-PCR using primers to specifically amplify the DUX4 ORF [19]. After electrophoresis (Figure 5A top panel) 1.7-kb (ORF, yellow arrow) and 2.1-kb (ORF with non-spliced introns) products were detected for the positive control (C2C12 cells transfected with pGEM42). These bands were present in samples derived from dFSHD myoblasts treated with the negative control AO, but much fainter or missing when these cells were treated with AOs against DUX4, and also missing in control myoblasts. On RNAs of aFSHD myoblasts, we performed an RT-qPCR with specific DUX4 primers (see Section Material and Methods) and observed the *DUX4* mRNA abundance was 9 times lower in cells treated with the AO (Figure 5A bottom panel). This residual *DUX4* mRNA abundance was similar to healthy control cells.

To validate that DUX4 inhibition prevented its target gene activation, we performed a RT-qPCR on TRIM43 mRNA as described [67] on the previously synthesized cDNA. As expected, TRIM43 mRNA abundance was decreased in cells transfected with the DUX4 AO or siRNA compared to non-transfected cells or cells treated with a negative control AO. This inhibition was similar between aFSHD and dFSHD cell cultures (Figure 5B).

In a different study, we found that DUX4c overexpression in proliferating myoblasts prevented their differentiation [37]. However, when DUX4c was induced after myoblasts had been switched to a differentiation medium they could fuse and formed myotubes of the dFSHD phenotype Our on-going studies indicate that inappropriate DUX4c activation as observed in FSHD muscles is a major contributor to the cytoskeleton anomalies observed in disorganized myotubes [81]. In aggregate these experiments indicate that DUX4c should be considered as an additional therapeutic target for FSHD besides DUX4.

### 3.4. Evaluation of AOs in Preclinical Models

The systematic evaluation in vivo of AOs targeting the *DUX4* mRNA has been hampered by the lack of a transgenic mouse model expressing DUX4 and presenting a myopathy (reviewed by [82]. The difficulty in generating these mice results from a combination of DUX4 toxicity, frequent leaks in its expression even from an inducible promoter, and its normal role in early embryogenesis.

To avoid these issues a transgenic mouse was developed with inducible PITX1 expression [83]. The Paired-like homeodomain transcription factor 1 (*PITX1)* gene is the first DUX4 transcriptional target that was identified [19]. A PMO was selected to interfere with protein synthesis and was complementary to a 25-nt sequence surrounding the translation start site of the *PITX1* mRNA. Cell penetration was favored by PMO conjugation to an octa-guanidinium dendrimer (vivo-PMO). PITX1 induction in skeletal muscles caused atrophy and weakness. The systemic delivery of the vivo-PMO (but not the PMO) at 10 mg/kg weekly for 6 weeks reduced PITX1 protein by 70%, reduced atrophic myofibers and improved muscle strength with no obvious sign of toxicity. This study provided a proof of principle that a vivo-PMO could decrease a pathogenic protein in vivo [83].

A model of FSHD muscle xenograft in an immunosuppressed mouse with electroporation-mediated PMO administration was successfully used to interfere with *DUX4* mRNA polyadenylation, but the system is very delicate to develop, and necessitates a supply of FSHD muscle biopsies that is obviously difficult to establish [74].

Another model is a local myopathy developed following injection of a recombinant AAV virus expressing DUX4 in a mouse hind limb: it was successfully used to co-inject an AAV expressing a siRNA and demonstrate *DUX4* mRNA silencing [57]. For its use in the AO evaluation the AAV had to be modified in order to include the *DUX4* 3’ UTR target, but the co-insertion of a DNA sequence encoding a V5 epitope tag at the end of the *DUX4* ORF generated artefactual splicing [84]. After muscle injection the repaired AAV vector did express DUX4-V5 protein that caused a local myopathy. We performed a pilot experiment in this model to test our lead AO interfering with intron II splicing in *DUX4* mRNA and that we had validated in primary FSHD myoblasts [27].

**Figure 6:**
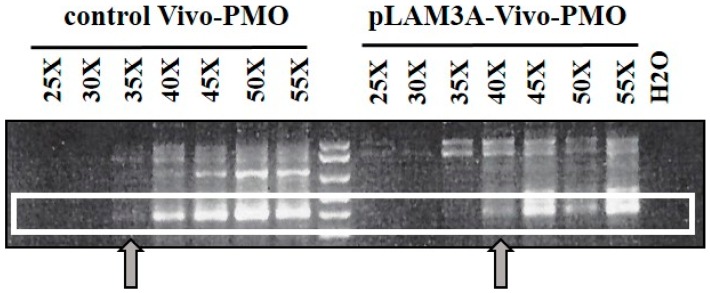
Evaluation of *DUX4* mRNA expression in mouse tibialis anterior muscles co-injected with rAAV-D4Z4/pLAM virus and either pLAM3A or control vivo-PMO. The TAs of the mice were injected with 1E11 DRP of D4Z4/pLAM AAV virus and 10 µg of vivo-PMO per leg. The mice were sacrificed 10 days post-injection, and total RNAs were extracted with Trizol. Reverse transcription was performed on 800 ng of DNase-treated total RNA with the 3’ adaptor of the RLM-RACE kit (Ambion) and 2 μL of the resulting cDNA were amplified by nested PCR for 50 cycles in total. The RT-PCR products were analysed by electrophoresis on a 1.5% agarose gel and stained with ethidium bromide.

Two mice were injected in the Tibialis anterior (TA) with rAAV-D4Z4/pLAM virus (described in [84]) and pLAM3A vivo-PMO (Figure 2) directed against the *DUX4* mRNA. For negative controls we similarly injected two mice with rAAV-D4Z4/pLAM and a vivo-PMO targeting human *beta-globin* mRNA. The mice were sacrificed 10 days later, their TAs collected, and total RNAs were extracted with Trizol. *DUX4* mRNAs were amplified by reverse transcription (RT) followed by a nested PCR (primer sequences in [84]). An electrophoresis of the RT-PCR products on agarose gel yielded a band at the expected 450-bp length for the TAs injected with control vivo-PMO (Figure 6). For the TAs injected with pLAM3A vivo-PMO the 450-bp band was present but at a lower intensity. In a semi-quantitative analysis this band could be detected after 35 PCR cycles for the TA treated with the control vivo-PMO but only after 40 cycles for the TA treated with pLAM3A vivo-PMO, suggesting a 30 (2^5^) fold difference in abundance. In contrast, when a similar experiment was performed with PMOs, no decrease in *DUX4* mRNA could be observed. Those preliminary data suggest that pLAM3A vivo-PMO prevents DUX4 toxic protein expression by causing *DUX4* mRNA degradation in vivo.

These data suggested that our successful inhibition of DUX4 expression with AOs in cell culture could translate to whole animal muscles and should be further evaluated in mouse models.

## 4. Conclusions

The encouraging clinical trials of PMOs in the treatment of young patients affected with DMD and their lack of toxicity ([64,85,86,87]) are raising hopes for the use of PMOS in FSHD. Several groups have independently developed AOs that target the *DUX4* mRNA and either destabilize it or prevent its translation to the toxic protein [27,71,74]. While no transgenic mouse model expressing DUX4 and showing signs of a myopathy were available in 2016, several research groups are developing new transgenic mouse models that appear much closer to the pathological presentation of FSHD and could potentially be used to evaluate AOs of different chemistries in vivo for efficacy, specificity and lack of toxicity.

The defects in DMD muscle membranes are considered to facilitate AO uptake, but it might be more difficult to target FSHD muscles for which no major membrane alteration has been described. Moreover, since DUX4 expression occurs in bursts in a low proportion of FSHD myonuclei [20,80], PMOs may need to be administered in large and repeated doses to achieve and maintain therapeutic efficacy, and this might result in deleterious secondary effects. Various chemical moieties have been added to PMOs in order to facilitate their membrane crossing and cell uptake (for examples, [88,89]) and some of these structures could be optimized for FSHD muscles. As well in the transgenic mouse with inducible PITX1 expression [83] and in a local myopathy induced by AAV-DUX4 injection [84] AOs with the vivo-morpholino chemistry could reduce the target mRNA levels while PMOs were not efficient. Further progress in DUX4-targeted therapeutic approaches for FSHD will also have to deal with DUX4 normal functions in non-muscle tissues such as testis and MSC. Moreover our data suggest that DUX4c might constitute another therapeutic target in FSHD [81].

## Supplementary Materials

The following are available online at www.mdpi.com/2073-4425/8/3/93/s1, Figure S1: Detection of the *DUX4* mRNAs in immortalized myoblast clones (FSHD1 and control) derived from a mosaic individual, Figure S2: Antisense oligonucleotides against *DUX4* mRNA decreased the number of DUX4-positive nuclei in primary FSHD myoblasts and prevented the development of the atrophic myotube phenotype.
