# Supplementary material for: Antisense Oligonucleotides Used to Target the DUX4 mRNA as Therapeutic Approaches in FaciosScapuloHumeral Muscular Dystrophy (FSHD)
Source: Genes (Basel). 2017 Mar 3;8(3):93. doi: 10.3390/genes8030093 (PMC5368697; doi:10.3390/genes8030093)
Supplement: Supplementary file 1 [file genes-08-00093-s001.docx]

**Supplemental data**

**
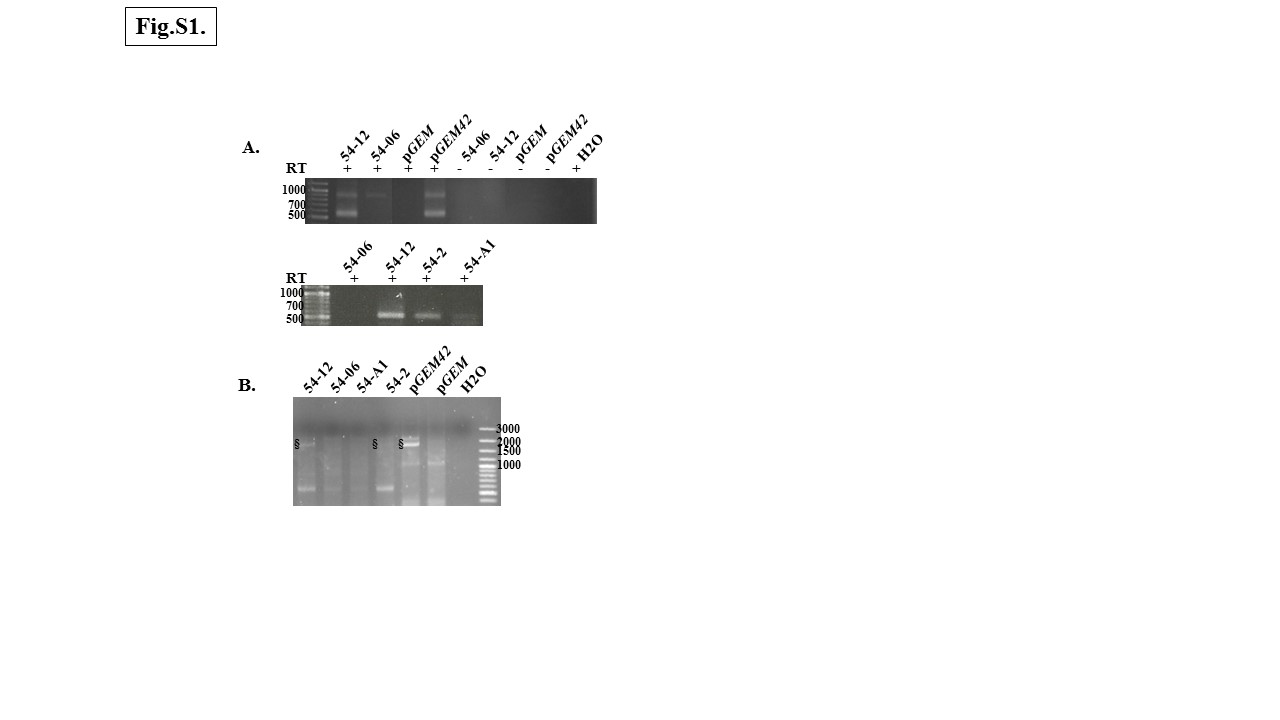
**

**Figure S1.** Detection of the *DUX4* mRNAs in immortalized myoblast clones (FSHD1 and control) derived from a mosaic individual. The cells were grown and differentiated for 5 days as described [67], and total RNA was extracted. (**A**) Reverse transcription (RT) was performed on 1 µg of DNase-treated total RNA with the 3′adaptator of the RLM-RACE kit (Ambion); 3 µl of the resulting cDNA were amplified by nested PCR (for details, see [27]). The 3’RACE products were analyzed by electrophoresis on a 1.5% agarose gel. The different products (400 bp; 550 bp and 900 bp) were sequenced and corresponded to the *DUX4* mRNAs with both introns spliced out, intron II spliced out and the unspliced mRNA, respectively. As RT-PCR controls we used total RNA from C2C12 mouse myoblasts transfected with the *pGEM* plasmid either without insert (negative control) or with a genomic fragment containing 2 D4Z4 units (*pGEM42*; [18]). The experiment was done in the presence (RT+) or absence (RT-) of retrotranscriptase to demonstrate the products did not result from amplification of contaminating genomic DNA. H_2_O: RT-PCR was performed with no RNA template. Clones 54-12, and 54-2 carry the FSHD1 genetic defect; clones 54-A1 and 54-6 have a normal size D4Z4 array (controls); (**B**) 2 µg of total RNA from (A) were reverse transcribed with a DUX4-specific primer and the resulting cDNA amplified by PCR as described [19]. The 1.7-kb product (§) was sequenced and corresponded to the *DUX4* mRNAs with intron II spliced out.


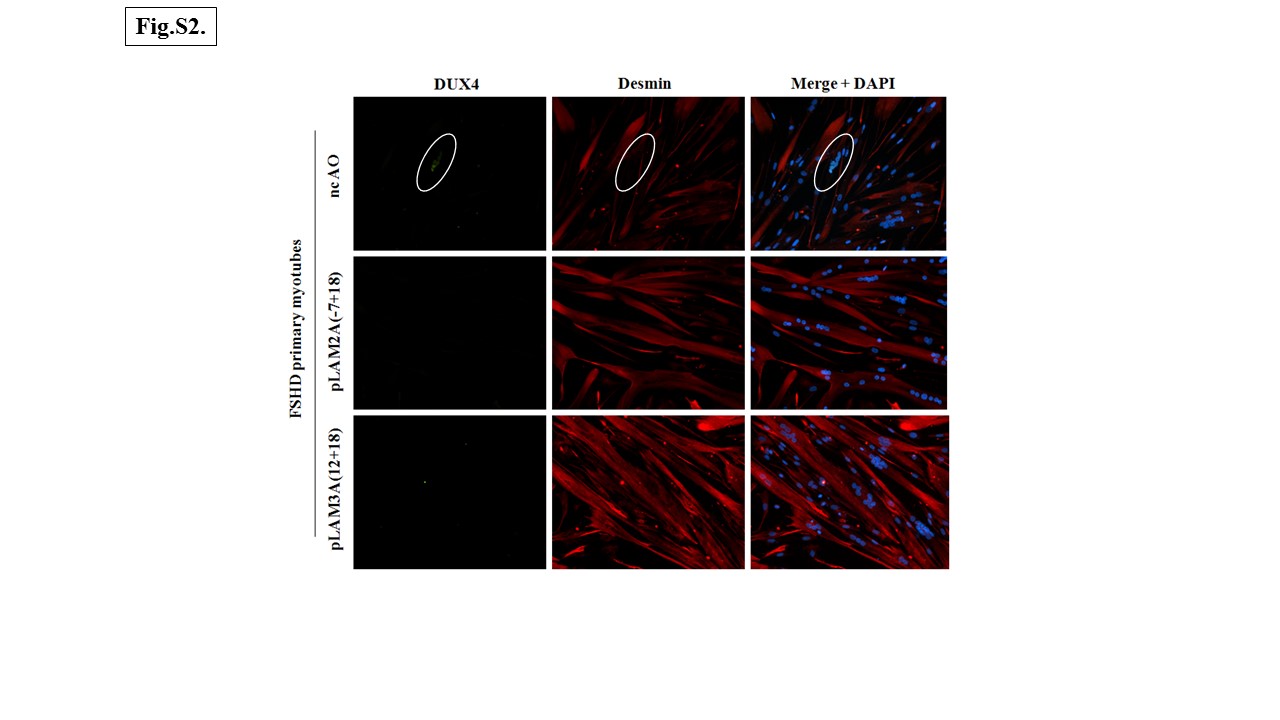


**Figure S2.** Antisense oligonucleotides against *DUX4* mRNA decreased the number of DUX4-positive nuclei in primary FSHD myoblasts and prevented the development of the atrophic myotube phenotype. 10^5^ primary FSHD myoblasts were seeded in 35 mm culture dishes. The next day, cells were transfected as described in Fig.3 with either the negative control AO (nc-AO, 600 nM) or the indicated AOs targeting the *DUX4* mRNA at optimized concentrations [27]. Differentiation was induced 4 hours after transfection. Eight days later cells were fixed in 4% PAF and incubated with 9A12 MAb directed against DUX4 (green) or anti-desmin (red) antibody (to confirm differentiation) and appropriate secondary antibodies coupled to Alexa Fluor dyes. The nuclei were labeled with DAPI and very few of them are DUX4-positive as expected [20, 80]. Based on desmin staining, the FSHD myotubes seemed larger following transfection with the AO against DUX4 than the nc-AO. Indeed the few DUX4-positive nuclei (circle, ncAO) are clustered in a very thin myotube similar to those where DUX4 causes cell death as observed by [34]. Such a difference also appeared on Figure 3 that corresponds to a representative field. This observation is in keeping with the inhibitory role of DUX4 in myoblast differentiation that would be suppressed with the *DUX4*-specific AO [25,28,32].
